# Control of antiferromagnetic domain distribution via polarization-dependent optical annealing

**DOI:** 10.1038/ncomms10720

**Published:** 2016-02-25

**Authors:** Takuya Higuchi, Makoto Kuwata-Gonokami

**Affiliations:** 1 Department of Physics, The University of Tokyo 7-3-1 Hongo, Bunkyo-ku, Tokyo 113-0033, Japan; 2 Photon Science Center, The University of Tokyo 7-3-1 Hongo, Bunkyo-ku, Tokyo 113-0086, Japan

## Abstract

The absence of net magnetization inside antiferromagnetic domains has made the control of their spatial distribution quite challenging. Here we experimentally demonstrate an optical method for controlling antiferromagnetic domain distributions in MnF_2_. Reduced crystalline symmetry can couple an order parameter with non-conjugate external stimuli. In the case of MnF_2_, time-reversal symmetry is macroscopically broken reflecting the different orientations of the two magnetic sublattices. Thus, it exhibits different absorption coefficients between two orthogonal linear polarizations below its antiferromagnetic transition temperature under an external magnetic field. Illumination with linearly polarized laser light under this condition selectively destructs the formation of a particular antiferromagnetic order via heating. As a result, the other antiferromagnetic order is favoured inside the laser spot, achieving spatially localized selection of an antiferromagnetic order. Applications to control of interface states at antiferromagnetic domain boundaries, exchange bias and control of spin currents are expected.

## Abstract (web summary)

The control of magnetic domains in antiferromagnets is limited by the lack of a net magnetic moment which may be manipulated by external stimuli. Here, the authors present an optical method for switching such domain states in MnF_2_ based on azimuth-dependent absorption of linearly polarized light.

## Graphical abstract

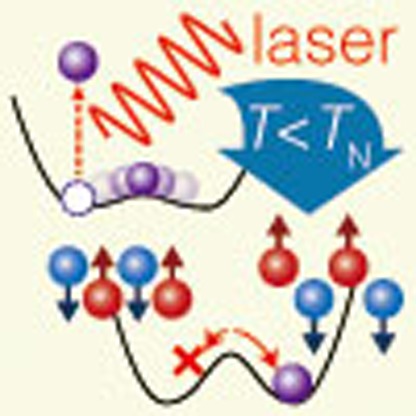

In antiferromagnetic materials, the magnetic moments of atoms or molecules, most likely produced due to electron spin, are aligned in a staggered manner. In the simple case of a two-sublattice antiferromagnet, there are two possible states: one sublattice is occupied by up spins and the other by down spins—or vice versa. This bistable magnetism is a manifestation of spontaneous symmetry breaking. As a result, macroscopic domains are formed depending on which antiferromagnetic state is realised, as is schematically depicted in Fig. 1a. The control of the spatial distribution of these antiferromagnetic domains is of particular importance^1^,^2^,^3^,^4^ because they determine the functionalities of antiferromagnetic materials with existing and potential applications, such as exchange coupling between adjacent antiferromagnetic and ferromagnetic orders^5^, dense non-volatile memory^6^,^7^ and conductivity by topologically protected metallic states confined to the antiferromagnetic domain boundaries^8^,^9^.

However, to date, such spatial control of antiferromagnetic domains remains challenging. A staggered magnetic field is a force correspondingly conjugate to antiferromagnetism, but generation of such a staggered field requires a flip in the sign of magnetic field on atomic length scales and is extremely difficult. Therefore, one needs to couple other macroscopic stimuli with the antiferromagnetic order for its control. This is not possible when the sublattices overlap each other by a translation of half a unit cell because a macroscopic stimulus cannot distinguish this microscopic translation that changes the sign of antiferromagnetic order parameter. Therefore, it is essential to break this symmetry for control of antiferromagnetic order other than the staggered field. For example, the linear magnetoelectric effect can couple electric field and antiferromagnetic order parameters, but its symmetrical requirement restricts its applications to particular materials^3^. Other than the linear magnetoelectric effect, control of the antiferromagnetic domains has been limited to methods employing indirect parameters, such as stress^10^,^11^ and magnetic field via higher-order magnetic susceptibilities^12^. Selection of an antiferromagnetic state over a whole crystal has been achieved by these methods, but the difficulty of local control of these macroscopic parameters has hindered spatial control of antiferromagnetic domain structures, and an external stimulus with a better control for local manipulation has been awaited. In this study, we employ the coupling between antiferromagnetic order and asymmetric optical absorption in an antiferromagnetic material without time-reversal symmetry.

Optical methods have provided unique opportunities in spatial control of (ferro-) magnetic domain structures. For example, demagnetization through optical absorption^13^ is a widely employed mechanism in magneto-optic recording for data storage. More recently, methods to control magnetic order through polarization-dependent light–magnetism interactions have been proposed and demonstrated^14^. Ferromagnetic domains are controlled via illumination with a circularly polarized light. The mechanism behind this phenomenon is considered to be combinations of various effects, such as the inverse Faraday effect^14^,^15^, the destruction of magnetic ordering through selective absorption due to magnetic circular dichroism (MCD)^16^,^17^ and optical spin pumping^18^. The timescales of these mechanisms vary, but one common feature in these mechanisms is that there are clear selection rules for the light–matter interaction, on the basis of the optical polarization state and the magnetic ordering parameter. Therefore, the magnetic ordering can be chosen by the polarization state of the pumping light, with the other conditions kept the same.

If a similar process employing optical polarization degrees of freedom directly coupled with antiferromagnetic order parameter is available, this will open the way for controlling the spatial distribution of antiferromagnetic domains, which is otherwise difficult as mentioned heretofore. However, to date, optical control of magnetic domains has been mainly achieved on the basis of selectivity of magnetic order through relations between the helicity of circularly polarized light and net magnetization; thus, it is not directly applicable to antiferromagnetic systems. Note that in hexagonal antiferromagnetic ScMnO_3_, linearly polarized light was found to induce photomagnetic instability of the antiferromagnetic orders having different spin orientations (a few degrees)^19^, but this is not applicable to select one of the two antiferromagnetic orders having opposite spin directions of two-sublattice antiferromagnet.

In this study, we propose a method for controlling antiferromagnetic order through optical annealing employing linearly polarized light. We demonstrate that illumination with linearly polarized light during field cooling of an antiferromagnetic material, MnF_2_, across its Neel temperature can determine the spatial distribution of antiferromagnetic states. This is possible when an antiferromagnetic material exhibits magnetic linear dichroism (MLD), the difference in optical absorption coefficients under a magnetic field between two cross-linear polarizations^20^,^21^,^22^. Such a coupling of an order parameter with non-conjugate external stimuli requires certain reduction of symmetry. For the MLD to exist, breaking macroscopic time-reversal symmetry is required, as discussed more in detail later. A noteworthy feature of the MLD is that it is odd with respect to the antiferromagnetic order parameter **L** (that is, the difference between sublattice magnetizations, **M**_1_−**M**_2_). Hence, as illustrated in Fig. 1a, one can selectively heat the domains of a particular magnetic order by illumination with linearly polarized light. When the crystal is cooled under this selective heating, the spins prefer the other antiferromagnetic state that is less exposed to the heat. We experimentally demonstrate that this scheme actually achieves selection of an antiferromagnetic state in MnF_2_, an antiferromagnetic insulator. By rotating the polarization azimuth angle by 90°, we can also choose the other antiferromagnetic state. Therefore, the desired antiferromagnetic state can be locally chosen by tuning the polarization azimuth of the illumination.

## Results

### Design of experiments for domain control with the MLD

**Figure 1:**
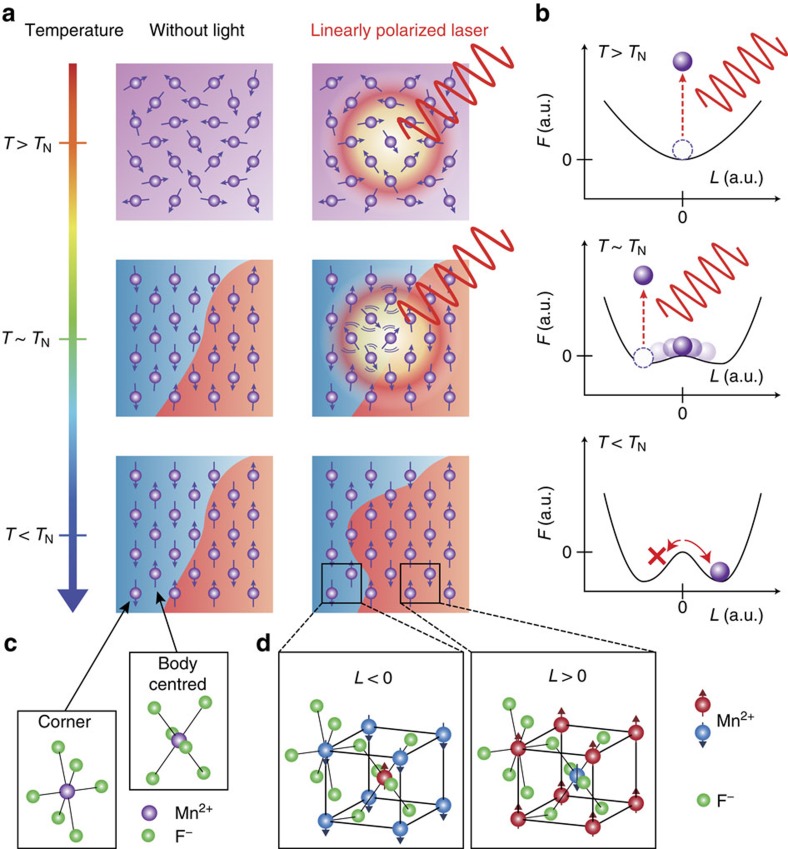
Schematics of the proposed method for selecting a particular antiferromagnetic state. (**a**) Schematic of the selective heating of undesired precursor antiferromagnetic order. The spins align antiferromagnetically below *T*_N_, and the domain distribution (red and blue areas) is determined by the residual stress and the spatial inhomogeneity of the magnetic field. When linearly polarized light illuminates the boundary in domain distribution during cooling, its absorption depends on the antiferromagnetic order through MLD, and thus it selectively destructs a particular antiferromagnetic order. As a result, the boundary of domain distribution is formed at a different position from that without illumination. (**b**) Schematic plots of how the Ginzburg–Landau energy *F* evolves as one cools down the temperature of the system. *F* has two degenerate minima below its Neel temperature, resulting in bistable orders. The purple ball shows the magnetic state, which prefers a low-energy state. The selective heating of particular antiferromagnetic order occurs below *T*∼*T*_N_, which prevents the system from entering into the antiferromagnetic state that has a larger optical absorption (that is, the left valley in this case), and the other order (the right valley) is chosen. (**c**) Schematics of two Mn^2+^ sites with different orientations of F^−^ ions surrounding the Mn^2+^. (**d**) Crystal structure of MnF_2_ with two possible antiferromagnetic orders having negative and positive *L*.

To find evidence that light can affect antiferromagnetic ordering, we focus on MnF_2_, an exemplary antiferromagnet. This crystal has two different Mn^2+^ sites at the corner and body centre in a unit cell, as schematically shown in Fig. 1b (ref. 23). Below its Neel temperature, these sites are occupied either by up and down spins or by down and up, resulting in two possible antiferromagnetic states. The spins in antiferromagnetic MnF_2_ align along the [001] axis; thus, **L** can be described as *L***e**_*z*_, where *L* is its amplitude and **e**_*z*_ is the unit vector along the [001] axis. Any spatial translation cannot overlap one order state to the other because the corner and body-centred sites have different fluorine environments: an additional rotation by 90° is required for this overlap. Therefore, the time-reversal symmetry is macroscopically broken^24^. This is important for applying optical methods to the control of antiferromagnetic order, because optical fields are macroscopic stimuli for the magnetic ordering; that is, the light cannot distinguish a half-unit-cell translation.

The time-reversal symmetry breaking in antiferromagnetic MnF_2_ manifests itself in the terms of the optical susceptibility tensor *χ*_ij_ that are odd with respect to **L**=*L***e**_*z*_, leading to MLD. Note that if time-reversal symmetry is maintained (that is, one order can overlap the other with a half-unit-cell translation), such odd terms cannot survive; **L** switches sign under this translation, but the macroscopic susceptibility should not. Of interest here within these terms that are odd to **L** are the ones that are also odd with respect to an external magnetic field, **B**=*B***e**_*z*_. Onsager's reciprocal relation^25^ provides *χ*_ij_(*L*,*B*)=*χ*_ji_(−*L*, −*B*). Therefore, the terms that are odd with respect to both *L* and *B* are symmetric with respect to the interexchange of subscripts^21^. These symmetric terms induce difference in the optical absorption coefficients of the two cross-linear polarizations (parallel to the [110] and [−110] axes in MnF_2_) under a magnetic field. This results in MLD. This is in contrast to the more commonly observed MCD, which originates from asymmetric off-diagonal terms that are odd with respect to only **B** (no contribution of **L**). The symmetric restriction for an antiferromagnetic material exhibiting MLD is the same as that of the piezomagnetic effect, which is allowed by 66 magnetic crystal classes^22^,^26^. Various other antiferromagnetic materials that break time-reversal symmetry also exhibit MLD and associated birefringence, such as CoF_2_ (ref. 20), Dy_3_Al_5_O_12_ (ref. 27), MnCO_3_, CoCO_3_ and CsMnF_3_ (ref. 28).

### Characterization of MLD

**Figure 2:**
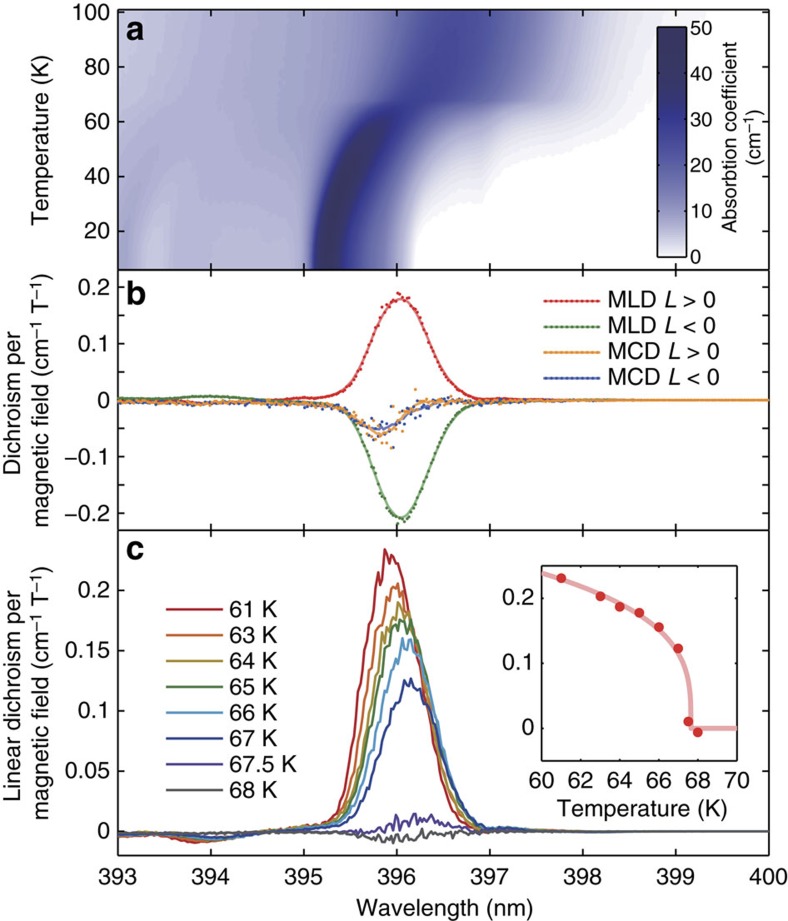
Optical and magneto-optical properties of MnF_2_ around its Neel temperature. (**a**) Temperature dependence of the absorption coefficient at *B*=0. (**b**) MLD and MCD spectra of MnF_2_ at 64 K, just below its Neel temperature. MLD is *α*_[−110]_(*B*)−*α*_[110]_(*B*) and MCD is *α*_L_(*B*)−*α*_R_(*B*), where *α* is the absorption coefficient and the subscripts denote the [−110]-linear, [110]-linear, left-circular and right-circular polarizations, respectively. They are both linear to *B* for a range of −5 T<*B*<5 T, and the slopes of the dichroisms per magnetic field strength are plotted. Dots are raw data, and smooth curves are moving averages over seven data points. (**c**) Temperature dependence of the MLD of the MnF_2_ crystal with *L*>0. Inset shows the peak amplitude of MLD derived by the fitting of the MLD spectra with Gaussian functions.

We first characterise the magneto-optical properties of MnF_2_ around its Neel temperature, *T*_N_=67.7 K. Figure 2a shows the temperature dependence of the absorption coefficient of MnF_2_ without an external field. The main peak corresponds to the magnon–exciton pair creation process^29^,^30^,^31^. Below *T*_N_, a prominent MLD is observed around this peak (Fig. 2b). The MLD changes its sign between the two antiferromagnetic order states prepared by a field-cooling method employing nonlinear magnetic susceptibility (see Methods for details). On the other hand, the standard MCD does not depend on the antiferromagnetic order. Figure 2c shows the temperature dependence of the MLD. The peak amplitude, *α*_MLD_(*T*), of the MLD shows a critical behaviour as a function of temperature *T*: *α*_MLD_ (*T*)=(*T*_N_−*T*)^*γ*^, *γ*=0.30±0.03. This critical exponent coincides with that of the antiferromagnetic order parameter^32^; this agreement strongly indicates that the MLD is proportional to *L*.

### Characterization of domain distribution

**Figure 3:**
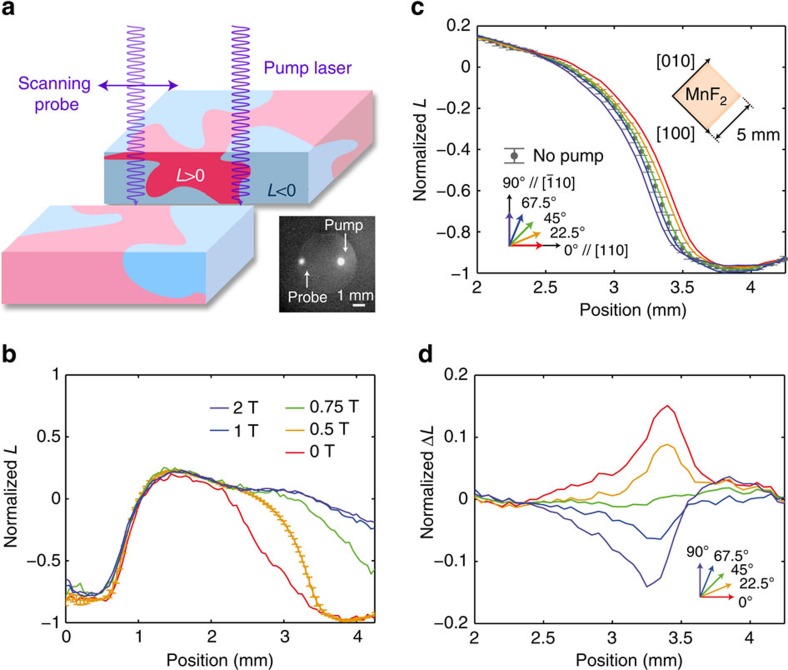
Experimental demonstration of optical control over the spatial distribution of antiferromagnetic states. (**a**) Schematic of the experiment, where the spatial distribution of antiferromagnetic states depends on the pump azimuth. Fluorescent image of the pump and probe beams are also shown. (**b**) Spatial distribution of *L* after cooling under various magnetic field values. The *L* values are normalized by that of a single antiferromagnetic domain. For the measurement with *B*=0.5 T, the *L* values are measured after three independent cooling processes and the mean values are plotted. Error bars show the s.d.'s among these three data sets. (**c**) Spatial distribution of *L* under various pump azimuthal angles. Dotted curve shows the baseline distribution without optical annealing and *B*=0.5 T. Error bars on the no-pump curve are same as in **b**. (**d**) Optically induced change in *L* from the baseline distribution.

On the basis of this well-characterized magneto-optical property of MnF_2_, we design an experiment to vary distributions of antiferromagnetic domains, as schematically illustrated in Fig. 3a. A continuous-wave laser is separated into pump and probe beams. By scanning the probe beam in one direction and measuring MLD (Fig. 3b), we obtain the one-dimensional spatial distribution of *L*. We compare the resultant distribution with and without optical pumping to extract the influence of light illumination. In this material, the size of the antiferromagnetic domains is typically between hundreds of micrometres and a few millimetres, as was previously observed by neutron topography measurements^33^,^34^, and is consistent with our results. To date, various other methods have been reported to observe spatial distribution of antiferromagnetic orders, such as polarized neutron topography^33^,^34^, second harmonic generation^35^, nonreciprocal reflection^36^,^37^ and X-ray diffraction^38^,^39^. Optical techniques such as the MLD have an advantage among them providing *in situ* information of spatial domain distribution. Even though the linear optical susceptibility of a bulk purely antiferromagnetic material alone does not contain terms that depends on the antiferromagnetic order parameter, higher-order susceptibility 35 or non-locality (change in wave number) in reflection^36^,^37^ introduces difference between them. In the case of the MLD, the order parameter sensitivity is introduced by an external magnetic field that perturbs the ideal, pure antiferromagnetic state.

Without the laser, the distributions of the antiferromagnetic order parameters are described using the nonlocal Ginzburg–Landau equation^40^ as follows:


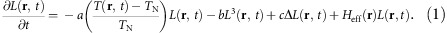


Here the first two terms are the Ginzburg–Landau energy according to the mean-field approximation, where *T*(**r**,*t*) and *L*(**r**,*t*) are local temperature and antiferromagnetic order parameter, respectively. *a* and *b* are parameters for describing the Ginzburg–Landau free energy. The third term describes the nonlocal interactions of the order parameter, and *c* is a parameter describing this non-locality. The last term includes the effective staggered field, *H*_eff_, mainly via magneto-elastic effects^10^,^11^,^41^ and higher-order magnetic susceptibilities^12^. Previous studies of choosing an antiferromagnetic state over a whole crystal of MnF_2_ were achieved by controlling this last term.

The distribution of *H*_eff_ is given by the residual stress, local impurities and weak transverse components of the magnetic field. Therefore, when we fix these parameters, the antiferromagnetic domain distributions are unchanged. Of these parameters, the external magnetic field is the only variable. We confirm that the domain distributions are robust for a fixed magnetic field. In particular, we cool down the sample three times under a magnetic field of 0.5 T (this condition is used for the following experiments) to see this reproducibility. The s.d.'s among these three experimental sets are shown as error bars in Fig. 3b. On the other hand, the magnetic field applied during the cooling changes the spatial distribution of the domains, as is shown in Fig. 3b. Note that the magnetic field is applied parallel to the ordered sublattice magnetization, and the magnitude of the field is 0.5 T and is much smaller than the spin-flop field of 11.5 T at the temperature (63 K) at which we characterized the domain distribution. The magnetization changes only around the vicinity of the spin-flop magnetic field (around ±2 T), and thus the induced magnetization at the field strength of 0.5 T can still be treated as a weak perturbation^42^,^43^. Indeed, the MLD showed linear dependence to the external magnetic field at around this magnetic field, which suggests that the external magnetic field is still sufficiently weak to be taken as a perturbation. On top, the induced magnetization alone does not break the degeneracy between the two possible antiferromagnetic orders because the magnetic field is parallel to the spin orientation axis. Even though the induced magnetization also causes ordinary MCD, it cannot distinguish antiferromagnetic order parameters and thus is irrelevant for this study.

### Control of domain distribution with the MLD

Next, we cool down the sample across *T*_N_ under spatially localized optical illumination. A magnetic field of 0.5 T is applied. The optical absorption coefficient depends on the antiferromagnetic order parameter and the polarization state of light due to the MLD. This can be described as a source of *L*-dependent heating in the thermal diffusion equation:


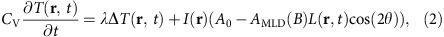


where *C*_V_ is the heat capacity, *λ* is the thermal conductivity and *I*(**r**) is the optical intensity. The second term describes the heat induced by the optical absorption. *A*_0_ is the absorption ratio of the light that does not depend on polarization. The polarization-dependent absorption ratio is −*A*_MLD_(*B*)*L*(**r**,t)cos(2*θ*), where *θ* is the polarization azimuthal angle measured from the [110] axis of MnF_2_. For linearly polarized light with *θ*=0°, heat by the polarization-dependent absorption in the domains with positive *L* is smaller than that in domains with negative *L*. Increases in temperature make the antiferromagnetic order unstable according to equation (1). Therefore, the domains with positive *L* are preferred because they are less heated. We experimentally observe this change in the distribution of antiferromagnetic domains, as shown in Fig. 3d. The region with positive *L* has a larger volume than that without pump light. On the other hand, light with the cross-linear polarization, that is, with *θ*=90°, is expected to affect the domain distributions oppositely, because the sign of the selective heating changes. As expected, the preference of the sign of *L* reverses when we rotate the polarization azimuth angle of the pump beam by 90°. We also observe the domain distribution for intermediate polarization azimuthal angles and obtain a systematic change. The systematic change is highlighted by subtracting the baseline domain distribution (that is, cooling under *B*=0.5 T without light) from the domain distributions after cooling under light illumination (Fig. 3d). The change in domain distribution is limited to the region where the light was illuminated.

### Numerical simulation of domain distribution

**Figure 4:**
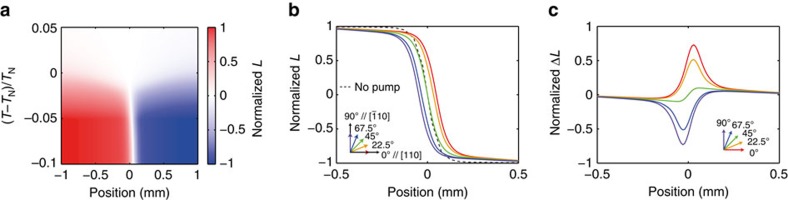
Simulated distribution of antiferromagnetic order parameters. (**a**) Temperature dependence of *L* along the *x* axis. Linearly polarized light with an azimuthal angle of 0 (that is, parallel to the *x* axis) illuminates the sample. (**b**) Spatial distribution of *L* at (*T*−*T*_N_)/*T*_N_=−0.1 after polarization-dependent optical annealing. The baseline distribution without optical pumping is also plotted. (**c**) Optically induced change in *L* from its baseline distribution without optical annealing.

To confirm the scenario that the domain distribution is determined via selective heating by light, we simulate the spatial distribution of antiferromagnetic order parameters according to equations (1) and (2). In this simulation, the sample is cooled down from its edges, which determines the boundary condition for the thermal diffusion equation (Fig. 4a). An effective staggered field, *H*_eff_(**r**), having a uniform gradient along the *x* axis, *H*_eff_(*x*)=*xH*_eff,0_, is assumed to emulate the creation of an antiferromagnetic domain boundary, which is achieved by considering a magnetic field slightly canted from the *z* axis. When the crystal is cooled down in the absence of light, the domain boundary is formed at *x*=0.

When a linearly polarized light is incident on the crystal, the resultant antiferromagnetic domain distribution after cooling differs from that without light. Figure 4a shows the temperature dependence of the domain distribution along the *x* axis, where optical polarization *θ*=0° is assumed. The domain with positive *L* has larger volume than the case without light (boundary at *x*=0). We simulate the polarization dependence of the domain distributions (Fig. 4b), as well as its difference from the baseline distribution (Fig. 4c). These simulated results show qualitative agreement with the experimental observations. The polarization dependence is well reproduced, and the spatial asymmetry in Δ*L* observed in experiment is also present in the simulation. Note that the spatial distribution of the domains appears blunter in the experiments. This is probably because the domain boundary was not formed parallel to the *z* axis and the boundary is likely to be formed with mosaic-like microdomain structures, and thus the optical probe integrated over the thickness of crystal provides a blurred distribution.

## Discussion

A further progress on the basis of this proof-of-concept study is envisioned. In the current study, the annealing process was carried out with control of the temperature of the whole crystal. It is attracting to replace this temperature control by local and fast heating, for example, via heating with pulsed lasers. Also, recent studies on lifetime of magnons in by means of neutron scattering suggested that there are mosaic-like smaller submicrometre domain structures MnF_2_ (ref. 44), which should exist at the boundary we observed. With our optical resolution (200 μm), these substructures are averaged out, and one can only see the vertically averaged distributions at larger scales, which is supported not only by the magnetic ordering but also by the other macroscopic effect such as the associated lattice distortion^45^. As the symmetry requirement for the selective heating does not rely on the domain size, but rather on the magnetic crystalline symmetry, it should be applicable to these micro domains as well. Higher optical resolution together with a sample thinner than a single domain is promising to address control of the microdomain structures.

In summary, we showed that illumination with linearly polarized light under an external magnetic field acts as a staggered stimulus to spins in antiferromagnetic MnF_2_ via selective heating. The linearly polarized light distinguished antiferromagnetic states through MLD, and its absorption heats the undesired state, thereby realising the desired state. With this method, we were able to select a particular antiferromagnetic state. The scope of our method is solely restricted by the symmetry of crystals that allow MLD (which is the same as the restriction of the piezomagnetic effect), which is found in various antiferromagnetic materials^22^,^26^. Therefore, numerous possible extensions are expected. For example, all-in/all-out-type antiferromagnetic materials, known as hosts of Weyl fermions^8^, satisfy this condition^39^,^46^, and possible control of their ordering provides unique opportunities in the growing field of topologically protected surface states.

## Methods

### Measurement of dichroism

In this study, we employed two experimental setups: one to measure the dichroism of a single antiferromagnetic domain and one to control the spatial distribution of antiferromagnetic orders. In both experiments, a single crystal of MnF_2_ with a [001] face, a thickness of 1 mm, and an area of 5 × 5 mm^2^ was placed in a sample holder in a cryostat (Microstat MO; Oxford Instruments). The cryostat had a superconducting magnet that generated a magnetic field from −5 to +5 T.

For measuring dichroism under a magnetic field, a light-emitting diode with a central wavelength of 397 nm and a bandwidth of 10 nm was employed as the light source. The light transmitted through the specimen was introduced into a monochromator (SpectraPro-300i; Acton Research) with a grating with a groove density of 2,400 G mm^−1^ and a blaze wavelength of 240 nm. Spectra of the transmitted light were recorded by a charge-coupled device-based optical multichannel analyser (OMA, LN/CCD-1,100 PG/UV; Princeton Instruments).

The difference in the absorption coefficients between the two orthogonal polarizations was measured as a function of the external magnetic field along the [001] axis. MLD is given by *α*_[−110]_(*B*)−*α*_[110]_(*B*) and MCD is given by *α*_L_(*B*)−*α*_R_(*B*), where *α* is the absorption coefficient and the subscripts denote the [−110]-linear, [110]-linear, left-circular and right-circular polarizations, respectively. These dichroisms showed linear dependence to the external magnetic field; thus, their slopes as functions of the field are plotted in Fig. 2b,c.

To compare the dichroism spectra of two possible antiferromagnetic orders, we needed to prepare one particular magnetic order over the entire crystal. This was achieved by cooling the crystal under a magnetic field slightly canted from the [001] axis (∼2°). We considered nonlinear terms in the free energy of the form *χ*_*ijkl*_ *L*_*i*_*B*_*j*_*B*_*k*_*B*_*l*_; here the terms *χ*_*zxxz*_=*χ*_*zyyz*_ are nonzero with respect to the magnetic point group of antiferromagnetic MnF_2_, 4‘/mmm'. These free-energy terms are odd with respect to the antiferromagnetic vector **L**. Therefore, canting the magnetic field from the [001] axis in the [110] direction resulted in additional differences in the forming energies of the two antiferromagnetic orders. This determined the antiferromagnetic order. This method to control antiferromagnetic order was employed by Kharchenko *et al*.^12^, and we employed the same technique.

### Observation and manipulation of antiferromagnetic domains

To observe and manipulate antiferromagnetic domains in MnF_2_, we employed a continuous-wave diode laser (Toptica) lasing at a wavelength of 396.25 nm selected by an external cavity with a grating. The laser beam was separated into probe and pump beams by a 10:90 separator. The weaker and stronger beams were the probe and pump beams, respectively. Both beams were focused onto the sample with spot sizes of ∼200 μm. The pump power was 13 mW and the light was linearly polarized. A half-wave plate was placed so as to control its polarization azimuth angle.

To increase the measurement sensitivity, the modulation method was employed for probing. The probe beam was sampled by a chopper with a frequency, *f*_ch_, of 200 Hz and modulated by a photoelastic modulator (PEM). In the PEM, a crystal oscillated at a frequency, *f*_PEM_, of 50 kHz, which changed the polarization state of the light with this frequency. The transmitted light was detected by a silicon photodiode, and the signal was analysed by three lock-in amplifiers having lock-in frequencies of *f*_ch_, *f*_PEM_ and 2*f*_PEM_, respectively. The signal for *f*_ch_ indicated the total transmitted intensity, and the 2*f*_PEM_ signal divided by *f*_ch_ was proportional to the MLD signal. The *f*_PEM_ signal contained information on both the MCD and the linear dichroism in the diagonal direction (in between [100] and [010]). This (non-magnetic) linear dichroism appeared when the probe beam was canted from a right angle to the sample surface that was useful for aligning the beam angle.

The positions of the pump and probe beams were observed by fluorescence from MnF_2_. When excitons were created by the laser, orange fluorescence was observed around a wavelength of 600 nm. To observe this fluorescence, a charge-coupled device camera was placed behind a coloured glass that filtered the laser. This fluorescence was useful for imaging because the emitted light was incoherent, and thus free from speckles.

If the magnetic field was canted from the [001] axis, its transverse component determined the antiferromagnetic order over the entire crystal, as was done in the first experiment. To make the crystal inhomogeneously contain both antiferromagnetic orders, we placed the crystal in the normal direction to reduce the effect of transverse magnetic fields. By matching the reflected beams from the sample surface and the window of the cryostat, this alignment was achieved within 0.1°.

### Numerical simulation

Solutions of the thermal diffusion equation and the non-local Ginzburg–Landau equation are simultaneously obtained via numerical integration using the Runge–Kutta method. A square crystal of MnF_2_ with an edge of 5 mm is discretized into a two-dimensional spatial mesh with a mesh size of 0.05 mm. The laser beam spot size is 0.1 mm in radius. Boundary conditions for the thermal diffusion equation are given by the temperature at the crystal edges, which decreases as a function of time to simulate the cooling of the crystal from its edges. The cooling rate is chosen to be slow enough so that the system undergoes an equilibrium state at the edge temperature during cooling. Values for the thermal diffusion constant and heat capacity of MnF_2_ are taken from refs 47, 48, respectively. The parameters *a* and *b* in the Ginzburg–Landau equation are taken from ref. 32. The non-locality parameter *c* of the magnetic order parameter is not known, and a best parameter is chosen to reproduce the experimental results.

## Additional information

**How to cite this article:** Higuchi, T. *et al*. Control of antiferromagnetic domain distribution via polarization-dependent optical annealing. *Nat. Commun.* 7:10720 doi:10.1038/ncomms10720 (2016).
